# A case of gastric gastrointestinal stromal tumour with two rare metastases after laparoscopic resection and imatinib therapy

**DOI:** 10.1093/jscr/rjag024

**Published:** 2026-01-30

**Authors:** Nathan J Bui, Hai T Bui

**Affiliations:** Ballarat Hospital, Grampians Health, 1 Drummond Street North, Ballarat Central 3350, Australia; Department of Upper Gastrointestinal and Hepato-Pancreato-Biliary Surgery, Footscray Hospital, Western Health, 89 Ballarat Road, Footscray, Melbourne 3011, Australia

**Keywords:** gastrointestinal stromal tumour, upper gastrointestinal surgery, chemotherapy

## Abstract

We report the case of a 70-year-old man with a gastric gastrointestinal stromal tumour who subsequently developed two rare metastatic recurrences to the subcutaneous tissue and skeletal muscle following initial curative resection and adjuvant imatinib therapy. This case illustrates the potential for atypical metastatic patterns in gastrointestinal stromal tumour and emphasizes the importance of long-term surveillance, even after extended disease-free intervals.

## Introduction

Gastrointestinal stromal tumour (GIST) is a rare mesenchymal neoplasm, accounting for ~1%–2% of primary gastrointestinal cancers [1]. The stomach is the most common primary site, followed by the small intestine. Metastatic spread most frequently involves the liver, omentum, and peritoneum [2]. We describe an unusual case of metastatic recurrence to the abdominal wall and tibialis posterior muscle several years after resection of a primary gastric GIST.

## Case report

**Figure 1:**
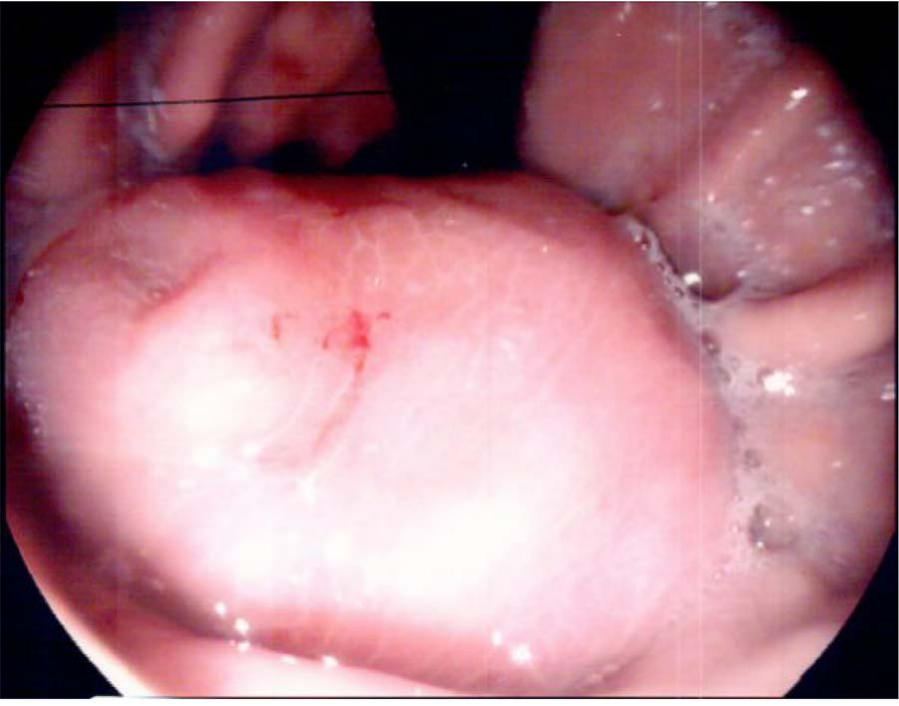
Gastroscopy identified a large submucosal mass in the gastric fundus.

A 70-year-old man initially presented with symptomatic anaemia. Following stabilization, outpatient abdominal computed tomography imaging identified a 10 cm soft tissue mass arising from the gastric fundus. Gastroscopy and biopsy confirmed a GIST (Fig. 1).

The patient underwent laparoscopic resection of the tumour. Histopathology demonstrated a spindle-cell neoplasm measuring 85 × 70 mm. This GIST’s immunohistochemistry markers was strongly positive for C-Kit (CD117) and CD34, focally positive for smooth muscle actin (SMA) and negative for S100. The mitotic index was ~20 per 50 high-power fields, consistent with a high-risk GIST. Adjuvant imatinib 400 mg daily was initiated and continued for 3 years. Surveillance imaging over the subsequent 6.5 years showed no recurrence.

**Figure 2:**
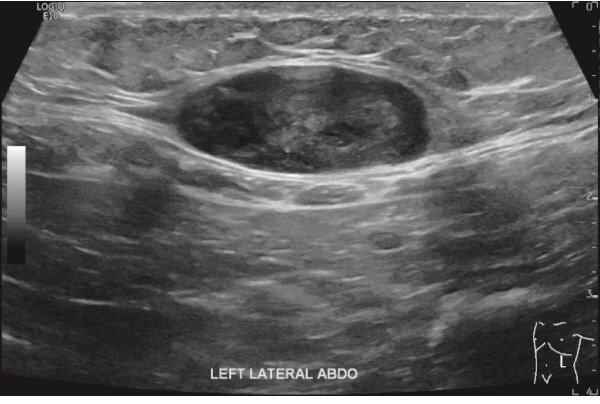
Abdominal US showed a well-defined soft tissue mass attached to the left inferior aspect of the rectus muscle.

However, 3.5 years after discontinuing imatinib, the patient identified a left upper quadrant subcutaneous mass at age 77. An abdominal ultrasound demonstrated a 16 mm hypoechoic lesion (Fig. 2). A subsequent biopsy confirmed metastatic GIST, again positive for C-Kit and CD34, negative for S100 and SMA, with a mitotic rate of 4 per 50 HPF. Initially suspected to represent a port-site metastasis, operative findings revealed a primary subcutaneous lesion, indicating haematogenous spread. The mass was fully excised and imatinib 400 mg daily was recommenced for a further 4 years, during which no recurrence occurred.

**Figure 3:**
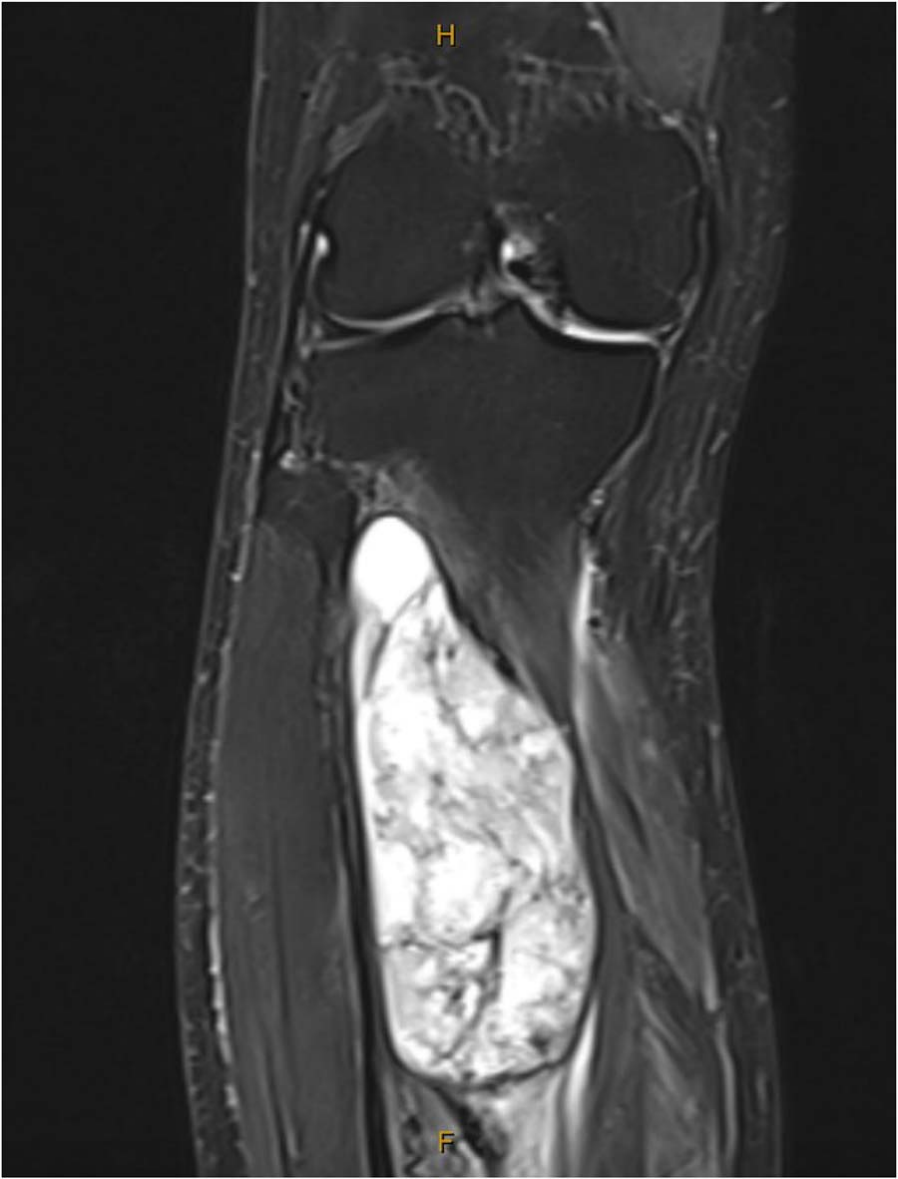
MRI showed a large tibialis posterior intramuscular lesion with proximal tibial involvement.

One year after the second discontinuation of imatinib, at age 82, the patient presented with progressive right leg swelling and pain. Magnetic resonance imaging (MRI) identified a 10 cm mass within the tibialis posterior muscle (Fig. 3). Biopsy confirmed metastatic GIST, positive for KIT, DOG1, and CD34. Imatinib therapy was restarted and adjuvant radiotherapy was under consideration at the time of reporting.

## Discussion

GISTs arise from the interstitial cells of Cajal, the pacemaker cells regulating gastrointestinal motility [2]. Patients typically present with gastrointestinal bleeding, abdominal pain, or a palpable mass, although some cases are detected incidentally [3].

Primary gastric GISTs generally have more favourable survival outcomes compared with those arising from the small bowel, colon, or rectum [4]. According to the American Joint Committee on Cancer 8th edition staging system, the primary tumour in this case corresponded to Stage IIIA disease. Both the Armed Forces Institute of Pathology and modified National Institutes of Health risk stratification models classified this tumour as high risk for recurrence or metastasis. Gastric GISTs >5 cm with >5 mitoses per 50 HPF are associated with an estimated 55% risk of disease progression [4]. This aligns with the subsequently observed extra-abdominal metastatic behaviour.

Current international guidelines for high risk GISTs recommend complete surgical resection followed by adjuvant imatinib 400 mg daily for at least 3 years in patients with high-risk GIST [5, 6]. Laparoscopic resection is considered oncologically equivalent to open surgery for tumours ≤10 cm, with benefits including reduced morbidity and shorter hospital stays [7].

Long-term surveillance for high risk GISTs is crucial.

- National Comprehensive Cancer Network recommends history, examination and CT, or MRI imaging every 3 months for 5 years, then annually [5].
- European Society for Medical Oncology suggests a CT or MRI scan every 3–6 months for the first 3 years during adjuvant therapy, then every 3 months for 2 years after cessation, then every 6 months until 5 years post-cessation and annually thereafter for 5 years [6].

Both recurrences in this case occurred after discontinuation of imatinib, reflecting a pattern of late relapse.

Metastases to subcutaneous tissue or skeletal muscle are exceedingly rare. One review identified only 10 cases of subcutaneous and 7 cases of skeletal muscle GIST metastases in the literature [8]. To our knowledge, this is the first reported case of sequential metastases to both sites following complete resection and adjuvant therapy for a gastric GIST.

Imatinib remains the standard therapy for recurrent or metastatic GIST. Treatment is generally recommended indefinitely, as interruption frequently leads to rapid disease progression [6]. In this patient, recurrence occurred after cessation of therapy on two separate occasions, supporting consideration of long-term or lifelong therapy in selected high-risk or previously relapsed patients.

## Conclusion

This case describes sequential subcutaneous and skeletal muscle metastases arising years after resection of a high-risk gastric GIST. The delayed recurrences following cessation of imatinib highlight the potential for late relapse and support consideration of prolonged or indefinite targeted therapy, as well as extended surveillance strategies. Clinicians should remain vigilant for atypical metastatic patterns, even many years after initial remission.
